# Overexpression of Ras Homologous C (RhoC) Induces Malignant Transformation of Hepatocytes In Vitro and in Nude Mouse Xenografts

**DOI:** 10.1371/journal.pone.0054493

**Published:** 2013-01-30

**Authors:** Shuli Xie, Mingguang Zhu, Guoyue Lv, Yajun Geng, Guofu Chen, Jian Ma, Guangyi Wang

**Affiliations:** 1 Department of General Surgery, the First Hospital of Jilin University, Changchun, Jilin, China; 2 Department of Immunology, School of Basic Medical Sciences, Jilin University, Changchun, Jilin, China; University of Hong Kong, Hong Kong

## Abstract

Ras homologous C (RhoC) is expressed in various cancers, including hepatocellular carcinoma (HCC). In this study, we first analyzed RhoC expression in 46 HCC tissue specimens and found that RhoC expression was significantly increased in HCC tissues compared to the adjacent normal liver tissues. Next, we investigated the role of RhoC in malignant transformation of normal hepatocytes. The HL7702 cell line was stably transfected with a RhoC expression vector and then subjected to cell proliferation, differentiation, colony formation, migration and invasion assays, as well as nude mouse xenograft assays. Gene expressions in these cells were determined using RT-PCR and Western blot. Overexpression of RhoC significantly promoted proliferation and anchorage-independent growth of HL7702 cells, but suppressed cell differentiation, as compared with the parental cells and the empty vector-transfected control cells. Moreover, RhoC overexpression induced migration and invasion of HL7702 cells *in vitro*. Molecularly, RhoC increased the expression of cell cycle-related genes, matrix metalloprotease 2 (MMP2), MMP9 and vascular endothelial growth factor (VEGF). In addition, RhoC-transfected cells formed tumors in nude mice, whereas vector-transfected HL7702 cells did not form any tumors in nude mice. This study demonstrated the role of RhoC overexpression in malignant transformation of normal human hepatocytes, suggesting that RhoC may function as an oncogene in hepatocytes.

## Introduction

Hepatocellular carcinoma (HCC) is a significant worldwide health problem, accounting for more than 600,000 cancer-related deaths annually in the world [1]. Approximately half of the HCC cases occur in China [2]. To date, the precise molecular mechanisms responsible for HCC carcinogenesis remain unknown. However, silence of tumor suppressor genes and activation of oncogenes, due to gene mutations, could contribute to hepatocarcinogenesis [3]. The risk factors that induce malignant transformation of normal hepatocytes include chronic infections of hepatitis B (HBV) and/or hepatitis C (HCV), aflatoxin toxin, cirrhosis, or consumption of large amounts of alcohol. These factors, especially HBV and HCV, can cause an autoimmune response to repeatedly attack liver cells. This constant cycle of damage followed by repair can lead to mutation of genomic DNA, and in turn lead to HCC development. A better understanding of the molecular mechanisms of HCC will help develop novel strategies for early detection, prevention, and therapy of this deadly disease.

Proteins of Rho GTPases belong to the Ras superfamily of small (∼21 kDa) signaling G proteins and regulate many aspects of intracellular actin dynamics. Rho proteins have been described as "molecular switches" and play an important role in cell growth, apoptosis, gene expression and carcinogenesis *in vitro, in vivo*, and in patients [4], [5]. These proteins are reported to regulate tumor formation, including proliferation, survival, invasion and metastasis [6]. The Rho subfamily consists of the highly conserved Ras homologous A (RhoA), RhoB and RhoC proteins. The expression of RhoA and RhoC is frequently induced in human cancers [7], [8]. Previous studies have provided evidence suggesting the critical role of RhoC in promoting progression of different cancers [4]–[8]. Our recent study demonstrated that RhoC was a key regulator of tumor cell growth and apoptosis in HCC cells [9]. These data suggest the potential involvement of RhoC in HCC carcinogenesis and progression. Thus, this study investigated the potential role of RhoC in mediating the malignant transformation of hepatocytes, which could provide insightful information into better understanding the mechanisms of HCC carcinogenesis.

## Materials and Methods

### Patient Specimens

A total of 46 patients who had undergone surgery for HCC at the Department of General Surgery, the First Hospital of Jilin University, were included. Fresh tissues were obtained immediately after surgical resection, including HCC tumor and adjacent normal liver tissues. The Ethics Committee of the First Hospital of Jilin University approved the protocol of this study.

### Immunohistochemistry

Paraffin-embedded tissue sections were prepared for immunohistochemical analysis of RhoC expression with an Ultra Sensitive TM SP kit according to manufacture’s instructions (Maxin Biotech Inc., Fuzhou, China). The samples were probed with an anti-RhoC primary antibody, and then an appropriate secondary antibody (Santa Cruz, USA). The antibody binding was visualized with 3, 3′-diaminobenzidine tetrahydrochloride (DAB). Two investigators who had no knowledge of the patients’ clinical status reviewed all of the immunostained sections. Cases with discrepant results were re-evaluated jointly until an agreement was reached. The degree of immunohistochemical staining was recorded using a semiquantitative and subjective grading system that considered both the intensity of staining and the proportion of tumor cells with an unequivocal positive reaction. Grades for stain intensity were: 0, no staining; 1, weak staining; 2, positive staining; and 3, strong staining. For rating stained areas: 0, positive staining in <5% of tumor cells; 1, positive staining in 5% to 25% of tumor cells; 2, positive staining in 25% to 50% of tumor cells; 3, positive staining in 50% to 75% of tumor cells; 4, positive staining in >75% of tumor cells. The staining index was calculated as the staining intensity multiplied by the positive area.

### Cell Lines and Culture

The normal human hepatocyte HL7702, human breast epithelial HBL-100 and fibroblast NIH 3T3 cell lines were obtained from the Shanghai Institute of Cell Biology at Chinese Academy of Sciences (Shanghai, China). Cells were routinely maintained in Dulbecco's Modified Eagle Medium (DMEM) containing 10% fetal bovine serum (FBS; all from GIBCO BRL, New York, USA) at 37 °C in a humidified incubator with 5% CO_2_ and 95% air. The growth medium was refed every 2 to 3 days and the subculture was carried out when cells reached 90% confluency using 0.25% trypsin.

### Plasmid Construction and Stable Gene Expression

To construct the expression vector carrying RhoC cDNA, we digested PMD-18T-RhoC (synthesized in our laboratory as previously described [10]) and pcDNA3.1 (Invitrogen, Carlsbad, CA, USA) plasmids with restriction enzymes *HindIII* and *EcoRI* (TAKARA). The released RhoC cDNA fragment was then ligated into the pcDNA3.1 plasmid to generate the pcDNA3.1-RhoC plasmid. After DNA amplification and sequence confirmation, the pcDNA3.1-RhoC vector was stably transfected into normal human hepatocyte HL7702 cells. The cells were divided into three groups: the control, the empty-vector control (EV control), and the pcDNA3-RhoC overexpression (RhoC OE) groups. For gene transfection, cells in the log-growth phase were seeded into cell culture dishes. Twenty-four hours later, the cells were transfected with the pcDNA3-RhoC plasmid or the pcDNA3 plasmid using Lipofectamine 2000 according to the manufacturer’s instructions (Invitrogen). The cells were then treated and maintained in G418-containing growth medium to generate stable RhoC-expressed HL7702 cells. Stable RhoC expression in the cells was then verified using reverse transcription polymerase chain reaction (RT-PCR) and Western blot.

### RNA Isolation and RT-PCR

Total cellular RNA was isolated using a Trizol reagent (Invitrogen) according to the manufacturer’s instruction. RNA was then reversely transcribed into cDNA using a RNA reverse kit (Takara, Dalian, China) according to the kit instructions. Next, PCR was performed to analyze gene expression, and α-tubulin was used as an internal control. Specific gene primers, such as Cyclin A, Cyclin G1, Cyclin D1, CDK4, p27, p27RF-Rho, matrix metalloprotease 2 (MMP2), MMP9, and VEGF, were synthesized by Sangon Biotech Co., Ltd. (Shanghai, China) (Table 1). PCR amplification conditions were as follows: denaturation at 94°C for 30 s, annealing at the gene-specific temperature for 30 s, and extension at 72°C for 1 min, for a total of 35 cycles. For α-tubulin, a total of 25 cycles was conducted. After gel electrophoresis in 1.5% agarose, ethidium bromide-stained bands were visualized by ultraviolet transillumination and the band intensity was quantified using Image Master software (CA, USA). Data were calculated from three independent experiments.

**Table 1:** PCR primer sequences, annealing temperature, and PCR product size.

Gene	Primers	Annealing temperature (°C)	PCR product size (bp)
RhoC	Forward: 5′-ATGGCTGCAATCCGAAAGAAG-3′	57	582
	Reverse: 5′-TCAGAGAATGGGACAGCCCCT-3′		
Cyclin G1	Forward: 5′-GTGTGTTGGACTGAGCTGCTTT-3′	58	592
	Reverse: 5′-TTCAGGAATTGTTGGAAGGTGAG-3′		
Cyclin A	Forward: 5′-GCATTGCAGCAGACGGCGCT-3′	62	555
	Reverse: 5′-TGGCTGTTTCTTCATGTAACCCAC-3′		
Cyclin D1	Forward: 5′-TGGATGCTGGAGGTCTGCGAG-3′	62	573
	Reverse: 5′-GGCTTCGATCTGCTCCTGGC-3′		
CDK4	Forward: 5′-GGGACCGTCAAGCTGGCTGA-3′	57	267
	Reverse: 5′-TCGAGGCCAGTCGTCTTCTG-3′		
P27	Forward: 5′-CGGGGTATGAAGAGCTTGCTTTGAT-3′	60	418
	Reverse: 5′-AACATTCAAAACTCCCAAGCACCTC-3′		
P27RF-Rho	Forward: 5′-TGTGACCGGAAGGGCTCCT-3′	57	580
	Reverse: 5′-GAGGAGAAGAGCTGTCCAAG-3′		
MMP-2	Forward: 5′-AACCCTCAGAGCCACCCCTA-3′	60	285
	Reverse: 5′-GTGCATACAAAGCAAACTGC-3′		
MMP-9	Forward: 5′-GGTGGACCGGATGTTCCC-3′	55	300
	Reverse: 5′-GCCCACCTCCACTCCTCC-3′		
VEGF	Forward: 5′-GGGCTGCTGCAATGACGA-3′	56	237
	Reverse: 5′-GTTTAACTCAAGCTGCCTCGC-3′		
α-tubulin	Forward: 5′-CACCCGTCTTCAGGGCTTCTTGGTTT-3′	57	527
	Reverse: 5′-CATTTCACCATCTGGTTGGCTGGCTC-3′		

### Protein Extraction and Western Blotting

Total cellular or nuclear protein was extracted for Western blotting as previously described with some modifications [11]. Briefly, total protein extracted from the human HL7702 hepatocytes was quantified using the BCA assay. A total of 30 µg of protein lysate was mixed with the loading buffer, denatured and then separated by 12% sodium-dodecyl sulfate polyacrylamide gel electrophoresis (SDS–PAGE). Then, proteins were transferred onto polyvinylidene fluoride (PVDF) membranes (Millipore, MA, USA), blocked with 5% w/v non-fat dry milk for 2 h, and then probed with primary antibodies at 4°C overnight. In the next day, the membranes were incubated with the secondary antibodies for 2 h at room temperature. Immunoreactive bands were detected with an enhanced chemiluminescence (ECL; Watson Biotechnology Co., Ltd., Beijing, China) according to manufacturer’s instructions. The bands observed on the films were analyzed with an automatic image analysis, and the integrated optical density (OPTDI) of each protein band was normalized to the OPTDI value of the corresponding β-actin band. Data were calculated from three independent experiments.

### Cell Proliferation and Differentiation Assays

Cell proliferation and differentiation were evaluated by silver nitrate staining and alkaline phosphatase staining, respectively. Briefly, for silver nitrate staining, cells were fixed with 95% ethanol and then stained with silver nitrate for 1 h at room temperature in the dark. After washing with deionized water, slides were dehydrated in a series of graded ethanol, cleared with dimethylbenzene and mounted with neutral balata. Black granules, which varied in size, were visualized under a light microscope. To quantify the data, we randomly selected three 20×microscopic fields, counted the black granules in each cell, and then the averaged number of black granules/total number of cells. For alkaline phosphatase staining, 95% ethanol-fixed cell samples were washed with distilled water, and then stained with 2% cobalt nitrate for 3–5 min at room temperature. After washing with distilled water, the samples were incubated with 2% fresh prepared (NH_4_)_2_S solution for 1–2 min and stained with 0.5% eosin for another 3–5 min. Finally, the samples were dehydrated, cleared and mounted. The activity of alkaline phosphatase in the cells was measured using an automatic biochemistry analyzer (Shenzhen, China) and the data were summarized as a percentage of the control in triplicate samples.

### Cell Migration and Invasion Assays

Cell migration and invasion assays were conducted using 8 µm polycarbonate filters in a Transwell chamber. For cell invasion, filters were coated with 60 µl of basement membrane Matrigel at a dilution of 1∶8 (50 mg/l). HL7702 cells were suspended in serum-free DMEM at a density of 5×10^5^ cells/ml, and then 200 µl of the cell suspension were added into the upper chamber of the Transwell chamber and 500 µl of the culture medium supernatants collected from NIH 3T3 cell cultures were added into the lower compartment of the Transwell chamber. After 24 h of incubation, the surface of the upper chamber cells was swabbed with cotton-swappers and the cells in the lower chamber were fixed with 4% paraformaldehyde (PFA) for 15 min, stained with violet for 30 min at 37°C, washed with distilled water and dried at room temperature. The number of migrated or invaded cells was counted under a phase contrast microscope.

### Soft Agar Colony Formation Assay

Twenty-four well plates contained a 0.8 ml base layer of 0.5% agarose in fully supplemented DMEM. Cells in the log phase were harvested, and then resuspended in culture medium and molten 0.5% agarose (to a final concentration of 0.3%). This cellular suspension (500 cells in 0.8 ml medium) was applied onto the base layer and allowed to set at room temperature. Cells were placed in the incubator at 37 °C with a humidified environment containing 5% CO_2_ and 95% air for 2 weeks. Clusters containing more than 50 cells or with a diameter over 75 µm was identified as a colony. The colony forming rate was determined by the following formula: colony forming rate = (the number of colonies/the number of total cells seeded) × 100%.

### *In vivo* Nude Mouse Xenograft Assay

A total of 10 nude mice were obtained from the Experimental Animal Center of the Chinese Academy of Medical Sciences, Beijing HFK Bio-Technology Co., Ltd. (Beijing, China). Animals were routinely housed in light (12 h dark/12 h light) and temperature-controlled rooms and had free access to food and water. Our animal protocol was approved by the First Hospital of Jilin University and the local Experimental Ethics Committee. All efforts were made to minimize animal suffering and to reduce the number of animals used.

For the nude mouse xenograft assay, 10 nude mice were randomly divided into two groups: the EV control group and the RhoC OE group. HL7702 cells stably expressing RhoC (PcDNA3-RhoC transfection group) or an empty vector (vector control group) were suspended in DMEM and adjusted to a density of 2.5×10^6^ cells/ml. Then, 0.2 ml of the cell suspension was injected subcutaneously into the back of the nude mice. The tumor volume and tumor weight was measured 4 weeks following inoculation.

### Statistical Analysis

Statistical analyses were performed using SPSS13.0 for Windows (SPSS Inc., Chicago, IL, USA.). Data were expressed as means ± standard deviation (SD) unless otherwise specified. Student’s t-tests were used to determine differences between two groups. Statistical significance was reached when *P*<0.05.

## Results

### Expression of RhoC Protein and its Clinical Relevance in HCC and Adjacent Normal Liver Tissues

**Figure 1:**
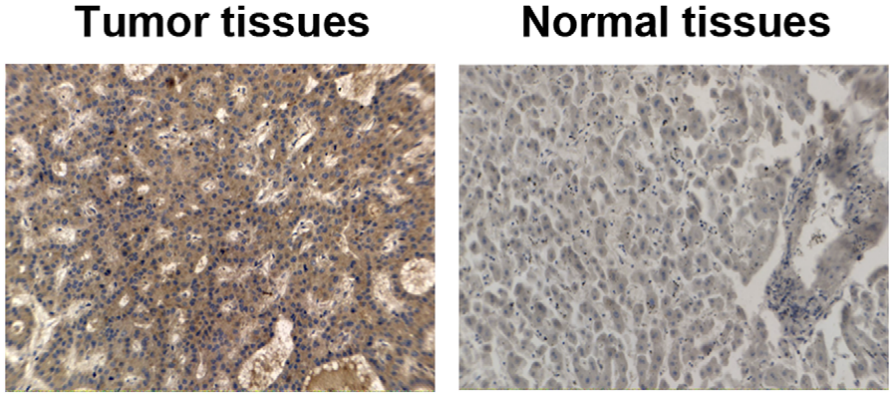
Expression of RhoC in HCC tissues and adjacent normal liver tissues. Tissue samples were obtained from HCC patients and immunostained with an anti-RhoC antibody. Cells with dark brown staining were positive. Magnification, ×20.

A total of 46 HCC patients were recruited for this study. RhoC expression was assessed immunohistochemically. Positive staining mainly distributed in the cytoplasm of both HCC and normal liver tissues (Fig. 1). The percentage of RhoC positive staining in HCC tissue was significantly higher than that of normal liver tissues (86.9%, 40 cases; 63.0%, 29 cases; *P*<0.05). As revealed in Table 2, 30 cases showed relatively strong RhoC immunoreactivity in tumor tissues (++, 18 cases; +++, 12 cases), whereas only 15 of these subjects showed strong RhoC expression in adjacent normal liver tissues (++, 15 cases; +++, 0), suggesting that the enhanced RhoC expression might be related with the tumorigenesis of HCC.

**Table 2:** Clinical relevance of RhoC expression in HCC tumor and adjacent normal liver tissues.

	*n*	RhoC immunoreactivity			
		−	+	++	+++
HCC tumor	46	6	10	18	12
Adjacent normal liver tissues	46	17	14	15	0

### Stable Expression of RhoC in Human HL7702 Hepatocytes

**Figure 2:**
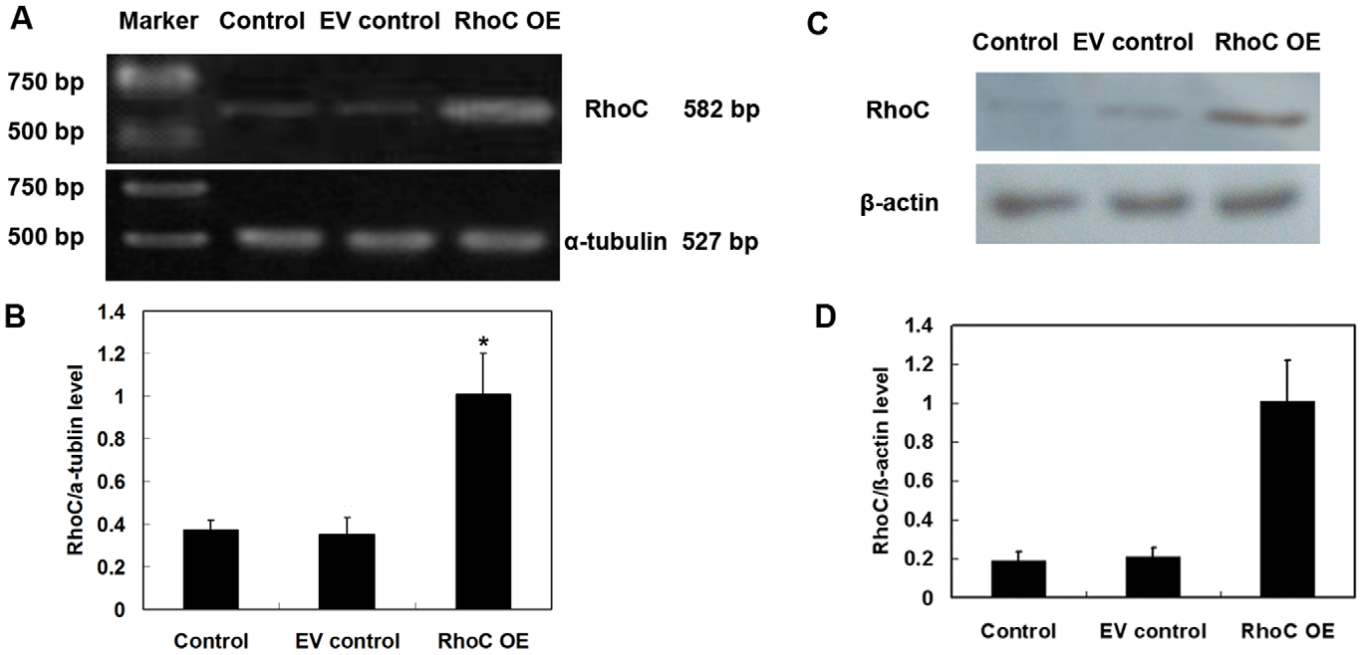
Stable expression of RhoC in human HL7702 hepatocytes. Cells were stably transfected with pcDNA3 or pcDNA3-RhoC and selected with G418-containing medium. Normal HL7702 cells were used as control. The expressions of RhoC mRNA and protein in each group were determined by RT-PCR (A, B) and Western blotting, respectively (C, D). Relative mRNA and protein levels of RhoC were quantified from three independent experiments (B, D). *P<0.05 compared with EV control. Note: EV control, vector-only transfected HL7702 cells; RhoC OE, RhoC-transfected HL7702 cells; Control, parental cells.

To investigate the potential effects of RhoC overexpression on promoting the malignant transformation of hepatocytes, we established a HL7702 cell line stably expressing RhoC, while empty vector-transfected HL7702 hepatocytes were used as negative controls. As shown in Fig. 2, RhoC mRNA and protein expressions were significantly elevated in stably RhoC-transfected cells (referred to as RhoC OE) as compared with those of the parental HL7702 cells (control) or cells transfected with the vector-transfected control cells (EV control) (all P<0.05). mRNA levels of the control, EV control and RhoC OE were 0.37±0.05, 0.35±0.08 and 1.01±0.19 (*P*<0.05). Protein levels of the control EV control and RhoC OE were 0.19±0.05, 0.21±0.05 and 1.01±0.21 (*P*<0.05).

### Effects of RhoC Overexpression on the Regulation of HL7702 Cell Proliferation and Colony Formation

**Figure 3:**
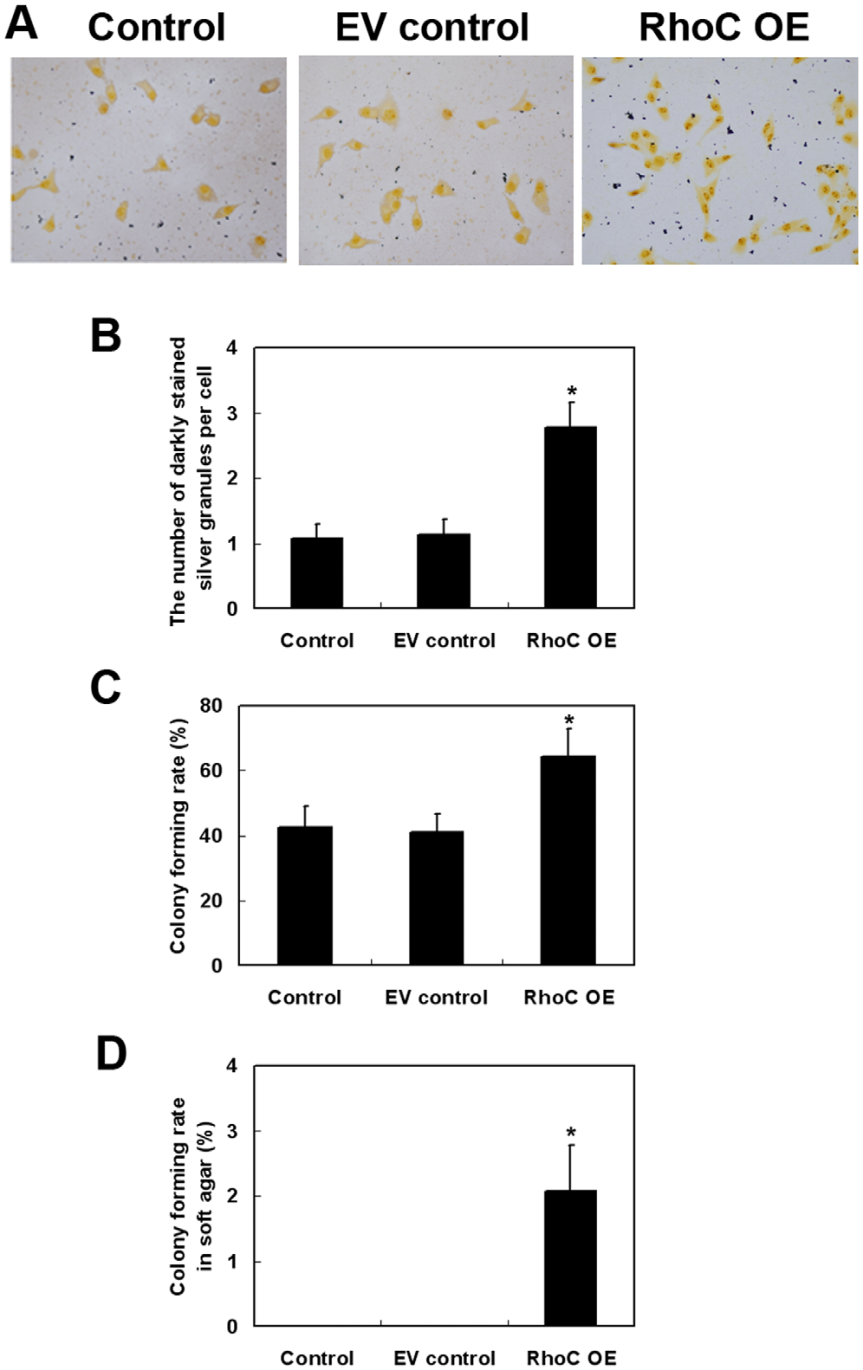
Effects of RhoC overexpression on promoting proliferation and colony formation of HL7702 cells. Silver nitrate staining analysis of cell proliferation capability (magnification: × 400; A) and quantitative data (B). For the colony formation assay, at least three microscopic fields were randomly selected and the number of darkly stained silver granules per cell was counted. The average number was presented. The colony formation rate in culture dishes (C) or in soft agar (D) was calculated from three independent experiments. *P<0.05 compared with EV control.

We next examined proliferation of HL7702 cells. Compared to parental and EV control cells, RhoC-transfected cells showed a greatly increased number of darkly stained silver granules in the cytoplasm of RhoC-modified HL7702 cells (*P*<0.05; Fig. 3A and 3B, respectively). Furthermore, the colony formation assay showed increased cell colonies in both culture dishes and soft agar (*P*<0.05 compared to parental control and EV control; Fig. 3C and 3D, respectively), suggesting that RhoC overexpression may promote anchorage-independent growth of human hepatocytes. In human breast epithelial HBL-100 cells transfected with RhoC gene, the cell proliferation was also promoted as compared with parental control and EV control (Fig. S1).

### Effects of RhoC Overexpression on the Regulation of HL7702 Cell Differentiation, Migration, and Invasion

**Figure 4:**
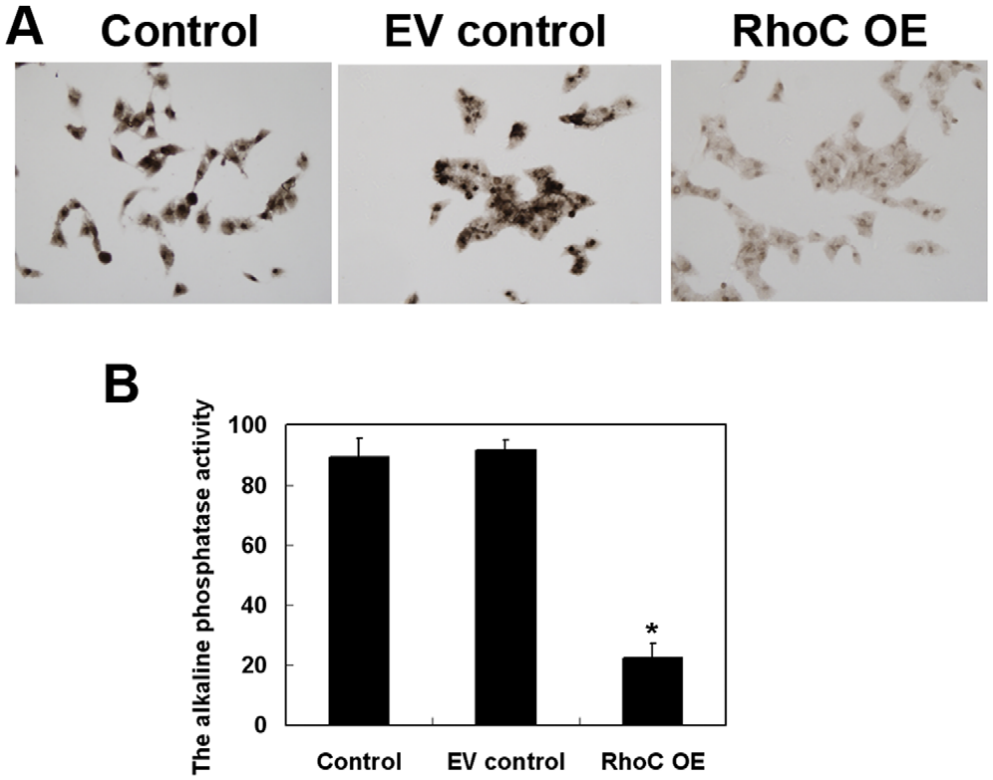
Effects of RhoC overexpression on the suppression of HL7702 cell differentiation. (A) Alkaline phosphatase staining (magnification, ×400). (B) Quantitative data. The alkaline phosphatase activity was determined and averaged from at least three randomly selected samples in each group. *P<0.05 compared with EV control.

We also determined the differentiation capability of RhoC-modified HL7702 cells using alkaline phosphatase staining. As shown in Figure 4, overexpression of RhoC dramatically reduced alkaline phosphatase activity as compared to the parental control and EV control cells (parental Control was 89.33±6.18; EV control, 91.33±3.40; RhoC OE, 22.33±4.92; *P*<0.05).

**Figure 5:**
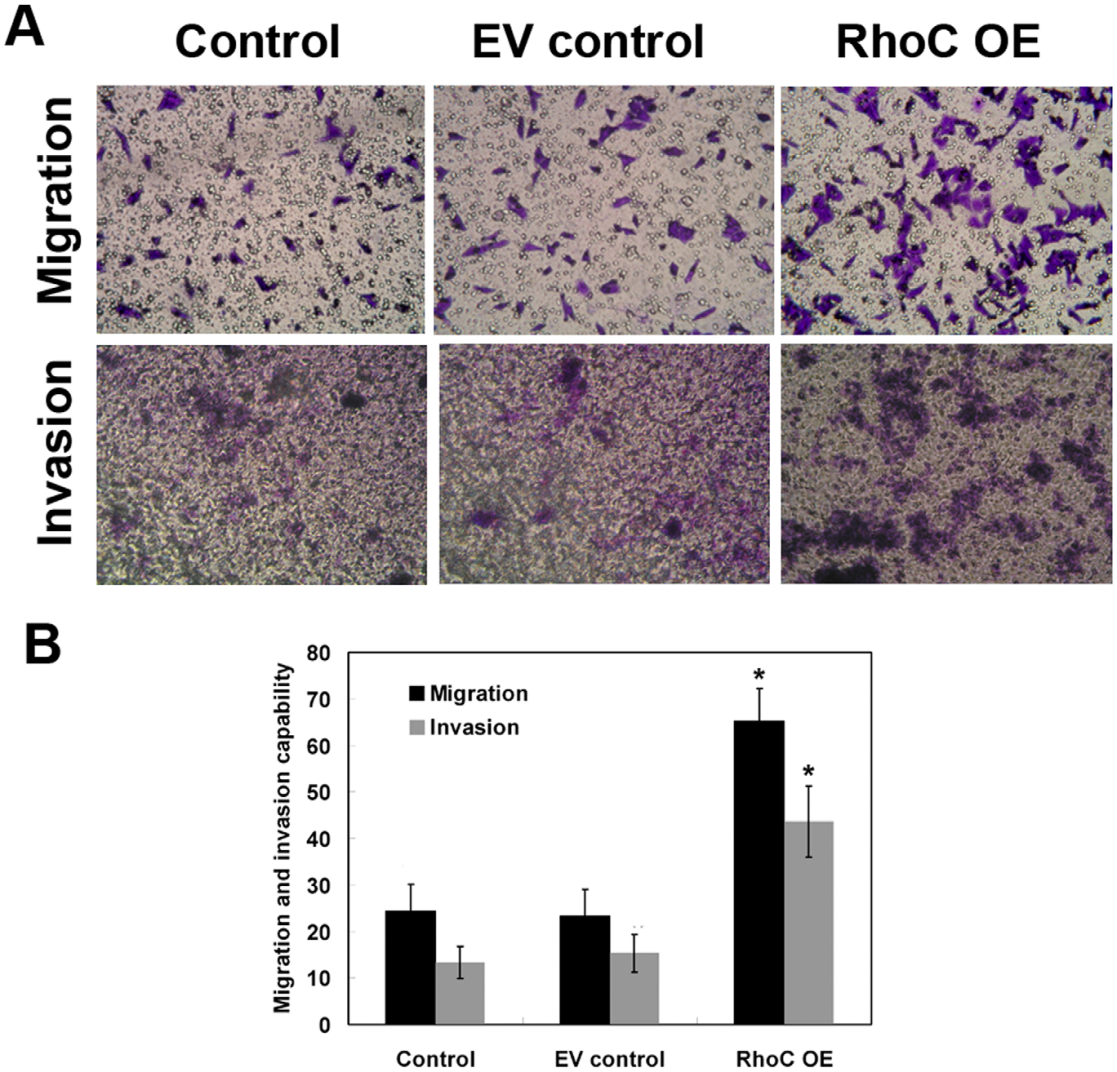
Effects of RhoC overexpression on promoting migration and invasion of HL7702 cells. (A) Cell migration and invasion assays. (B) Quantitative data. The percentage of migrated and invaded cells was determined and averaged from at least three randomly selected fields. *P<0.05 compared with EV control.

Next, we assessed the migration and invasion ability of HL7702 cells after RhoC modification. We found that RhoC overexpression remarkably promoted cell migration and invasion (Fig. 5).

### Effects of RhoC Overexpression on the Regulation of Cell-growth- and Invasion-related Genes in HL7702 Cells

**Figure 6:**
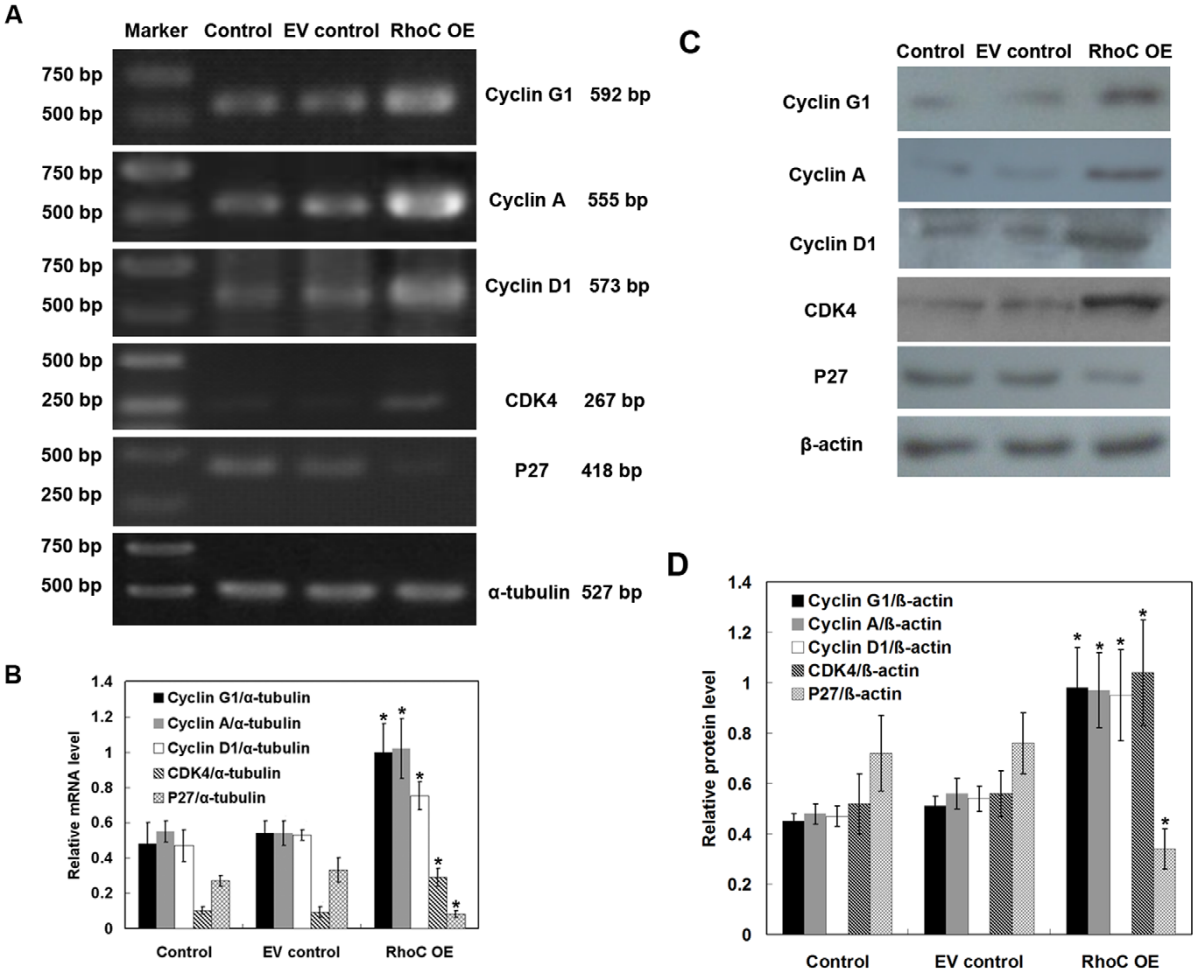
Effects of RhoC overexpression on the regulation of cell cycle-related gene expressions. (A) RT-PCR. The expression of Cyclin G1, Cyclin A, Cyclin D1, CDK4, and p27 mRNA in HL7702 cells was determined by RT-PCR. α-tubulin was used as an internal control. (B) Quantitative data. The relative mRNA expression of the target gene was quantified from three independent experiments. (C) Western blotting. The expression of Cyclin G1, Cyclin A, Cyclin D1, CDK4, and p27 proteins in HL7702 cells was determined by Western blotting. β-actin was used as an internal control. (D) Quantitative data. The relative protein expression of target gene was quantified from three independent experiments. *P<0.05 compared with EV control.

**Figure 7:**
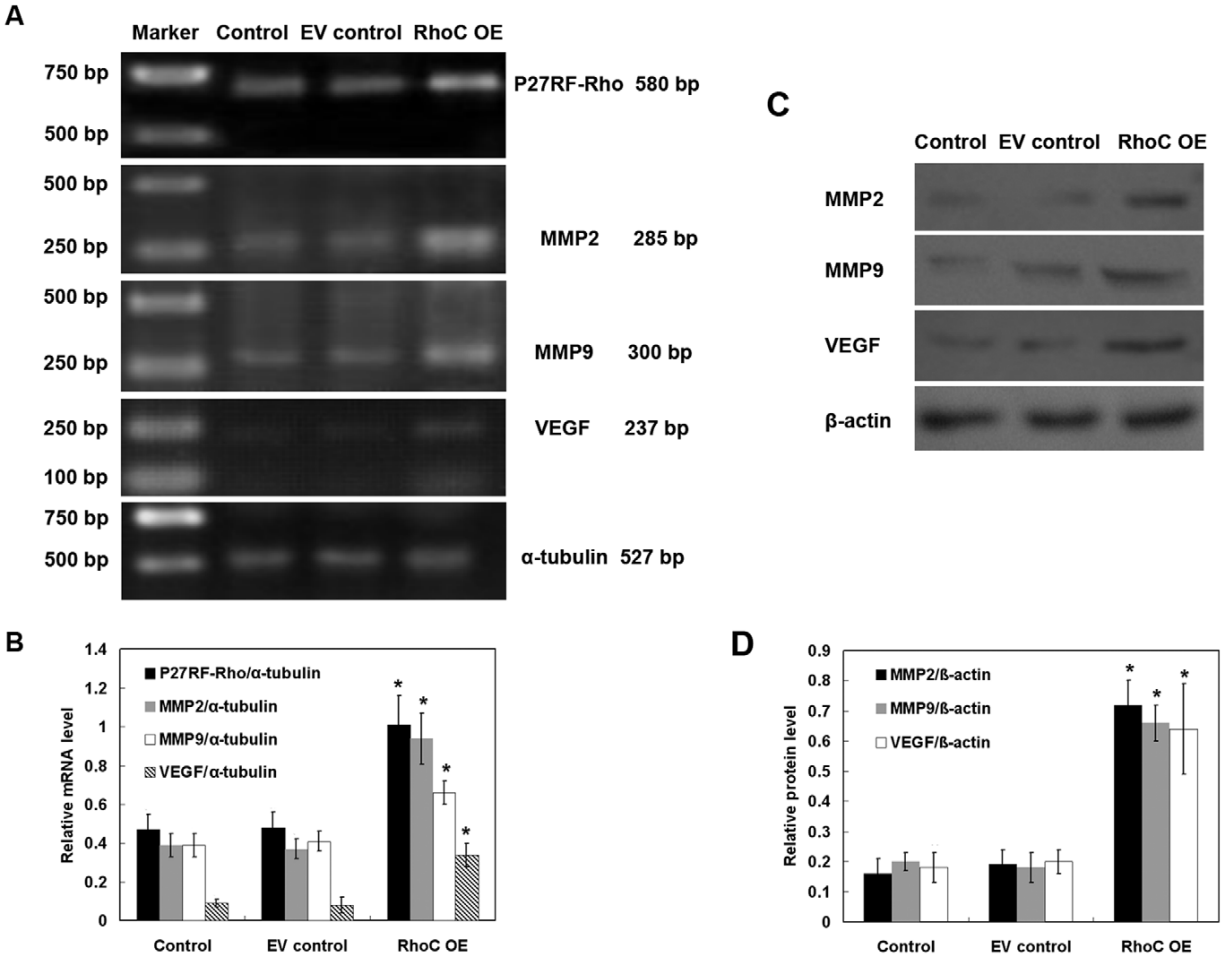
Effects of RhoC overexpression on the regulation of cell migration- and invasion-related gene expressions. (A) RT-PCR. The expression of P27RF-Rho, MMP2, MMP9 and VEGF mRNA in HL7702 cells was determined by RT-PCR. α-tubulin was used as an internal control. (B) Quantitative data. The relative mRNA expression of the target gene was quantified from three independent experiments. (C) Western blotting. The expression of MMP2, MMP9 and VEGF proteins in HL7702 cells was determined by Western blotting. β-actin was used as an internal control. (D) Quantitative data. The relative protein expression of the target gene was quantified from three independent experiments. *P<0.05 compared with EV control.

We further determined expression of cell growth-, migration- and invasion-associated genes using RT-PCR and Western blotting in HL7702 cells. RhoC overexpression significantly upregulated expressions of cell cycle-related genes, such as Cyclin G1, Cyclin A, Cyclin D1 and CDK4, and down-regulated the mRNA expression of p27, which is an inhibitor of G1 cyclin-CKD protein kinase (Fig. 6), indicating that RhoC may mediate cell proliferation by regulating the expressions of cell cycle-related genes. Moreover, compared to the parental control and EV control cells, P27RF-Rho, MMP2, MMP9 and VEGF mRNA and protein expressions were dramatically increased in cells stably expressing RhoC (all *P*<0.05; Fig. 7), indicating that RhoC promotes the migration and invasion of HL7702 cells by expression of these genes. Similar results were obtained in human breast epithelial HBL-100 cells transfected with RhoC (Fig. S2).

### Overexpression of RhoC Enhanced the Tumorigenicity of HL7702 Cells *in vivo*

**Figure 8:**
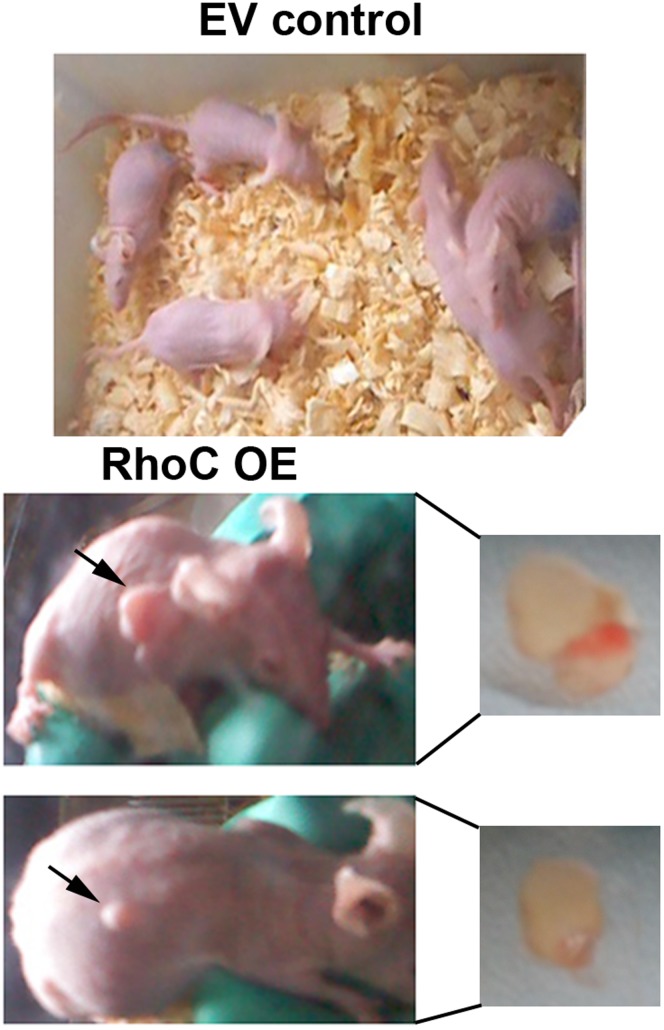
Nude mouse xenograft assay. Nude mice were inoculated with cells stably expressing either an empty vector (EV control) or pcDNA3-RhoC (RhoC OE). Four weeks later, tumor formation was not found in the EV control group, whereas two mice had tumor formation in RhoC OE mice (n = 5 for each group). Arrows indicate locations of tumor formation.

To determine the tumorigenicity of RhoC-modified HL7702 cells *in vivo*, we performed nude mouse xenograft assays. None of the control mice had tumor formation 4 weeks after subcutaneously injecting empty vector-transfected HL7702 cells (Fig. 8). In contrast, two mice (n = 5) exhibited obvious tumor formation after inoculation of HL7702 cells stably expressing RhoC. The tumor volume was 0.91 cm^3^ and 2.56 cm^3^, respectively, and the tumor weight was 0.62 g and 1.74 g, respectively. These data indicate that overexpression of RhoC can enhance the tumorigenicity of HL7702 cells in nude mice.

## Discussion

HCC is a common malignancy and most prominent in China. However, the molecular mechanism of hepatocarcinogenesis has not been fully elucidated [12], [13]. In general, malignant transformation is thought to be caused by abnormal gene expression essential to cellular processes, such as cell cycle control, cell growth, differentiation, apoptosis and adhesion, or other functions at the cellular, molecular, and genetic levels [14]. It has been suggested that the activity of Rho family members undergoes time- and spatial-dependent fluctuations during the different steps involved in tumor progression and metastasis [6]. Moreover, studies indicate that Rho-protein-dependent cell signaling is important for malignant transformation [15], [16], [17]. Our current study further demonstrated the effects of RhoC overexpression on promoting HL7702 transformation to malignant cells.

Previously, overexpression of RhoC protein has been detected in inflammatory breast cancer (IBC), an aggressive form of breast cancer that is highly infiltrative and metastatic with poor prognosis of patients [18]. Expression of RhoC mRNA levels in tumor tissues collected from HCC patients were significantly higher than that of the para-cancerous normal liver tissues [19]. Other studies showed that RhoC enhanced proliferation of tumor cells, including esophageal squamous cell carcinoma [20] and gastric carcinoma [21]. In contrast, knockdown of RhoC expression efficiently inhibited tumor cell proliferation [21], [22]. Our recent study demonstrated that knockdown of RhoC in HCC cells by RNA interference significantly decreased cell proliferation, induced cell apoptosis, reduced cell invasion and migration, elevated cell differentiation, increased cell membrane permeability, and impaired mitochondrial function [9]. Here, we found that overexpression of RhoC induced malignant transformation of normal hepatocytes *in vitro* and tumorigenicity *in vivo*. Thus, genetic modification of normal cells offers a valuable tool for understanding oncogenesis [23].

Among various members of the Rho family proteins, RhoC is suggested to be dispensable for embryogenesis and tumor initiation but essential for tumor metastasis [24], [25]. Thus, expression of RhoC protein in metastatic regions of pancreas cancer was reported to be higher than that of the primary tumor lesions [26]. In addition, RhoC is associated with cancer invasion in melanoma [24], IBC [27] and ovarian cancer [28]. Genome-wide analysis of gene expression also indicated that RhoC gene was involved in vascular invasiveness of HCC [29]. Our current data confirmed these findings.

Furthermore, the cell cycle regulator Cyclin D1 can serve as a proto-oncogene, and Cyclin D1 overexpression is the hallmark of malignant transformation in mantle cell lymphoma (MCL) [30]. High levels of Cyclin A expression have been demonstrated to associate with higher tumor grade and poor prognosis of breast cancer [31]. Cyclin G overexpression is a frequent event in colorectal cancer [32]. Moreover, CDK4 is a cell cycle regulator involved in early G1 cell cycle progression and has a direct role in angiogenesis *in vivo* [33]. Reduced expression of the cell cycle inhibitor p27 was associated with malignant transformation of the ovarian epithelium and FIGO stage [34]. Consistent with these observations, our current data showed that overexpression of RhoC significantly increased mRNA expressions of Cyclin D1, Cyclin A, Cyclin G and CDK4 mRNA, and decreased the level of p27 mRNA in stable RhoC-overexpressed hepatocytes. These findings indicate that RhoC may promote malignant transformation by enhancing uncontrolled proliferation of normal hepatocytes. In addition, MMP2 and MMP9 enzymes play an essential role in cell-matrix interaction and tumor invasion and migration [35]. VEGF has been well-documented to play a role in tumor angiogenesis [36]. Again, Hoshino et al. recently reported that p27RF-Rho, a p27(kip1)-binding protein, promoted cancer metastasis via activation of RhoA and RhoC [37]. In accordance with previous studies, our current study showed that the expression of P27RF-Rho, MMP2, MMP9, and VEGF mRNA was dramatically increased in cells stably expressing RhoC, as compared with the controls, indicating that RhoC promoted the migration and invasion of HL7702 cells.

Although the underlying mechanism of RhoC overexpression-induced malignant transformation of normal hepatocytes remains to be elucidated, a variety of mediators and signal pathways may contribute to this process. For example, formin family proteins, such as formin-like 2 (FMNL2) and FMNL3, have been identified as specific RhoC effectors and contribute to RhoC-modulated invasive cell motility [38], [39]. In addition, overexpression of RhoC promotes human melanoma cell invasion via the phosphatidylinositol-3 kinase (PI3K)/Akt signaling pathway [40]. In prostate cancer, RhoC promotes tumor metastasis by sequential activation of Pyk2, focal adhesion kinase (FAK), mitogen-activated protein kinase (MAPK) and Akt, followed by the upregulation of MMP2 and MMP9, resulting in the induction of tumor cell invasion [41]. However, our current study did not investigate these pathways and future study will continue to explore the potential downstream effectors of RhoC during malignant transformation. Finally, the nude mouse xenograft assays, in our study, showed that only 2/5 (40%) mice had tumor formation after inoculation with RhoC-transfected HL7702 cells, for a reason that is not clear. In future studies, we will verify these data using a different background of mice or use inducible gene expression vectors.

In summary, our present study demonstrated that RhoC overexpression induces malignant transformation of normal human hepatocytes by promoting cell proliferation, migration, and invasion. These findings imply that RhoC may serve as a novel oncogene during malignant transformation of hepatocytes. Accumulating evidences highlight the critical role of RhoC in tumor metastasis [25], [27], [42], and our current study provide valuable insights into understanding the complex actions of RhoC in regulating biological activities of tumors.

## Supporting Information

Figure S1 **Growth curve of human breast epithelial HBL-100 cells transfected with empty vector or RhoC.** Cells without transfection were applied as control.
(TIF)

Figure S2 **Effects of RhoC overexpression on the regulation of cell migration- and invasion-related gene expressions in human breast epithelial HBL-100 cells.** (A) RT-PCR. The expression of MMP2, MMP9 and VEGF mRNA in HBL-100 cells was determined by RT-PCR. α-tubulin was used as an internal control. (B) Quantitative data. The relative mRNA expression of target gene was quantified from three independent experiments. (C) Western blotting. The expression of MMP2, MMP9 and VEGF proteins in HBL-100 cells was determined by Western blotting. β-actin was used as an internal control. (D) Quantitative data. The relative protein expression of target gene was quantified from three independent experiments. *P<0.05 compared with EV control.
(TIF)
