# Supplementary figures and images for: Overexpression of Ras Homologous C (RhoC) Induces Malignant Transformation of Hepatocytes In Vitro and in Nude Mouse Xenografts
Source: PLoS One. 2013 Jan 30;8(1):e54493. doi: 10.1371/journal.pone.0054493 (PMC3559837; doi:10.1371/journal.pone.0054493)

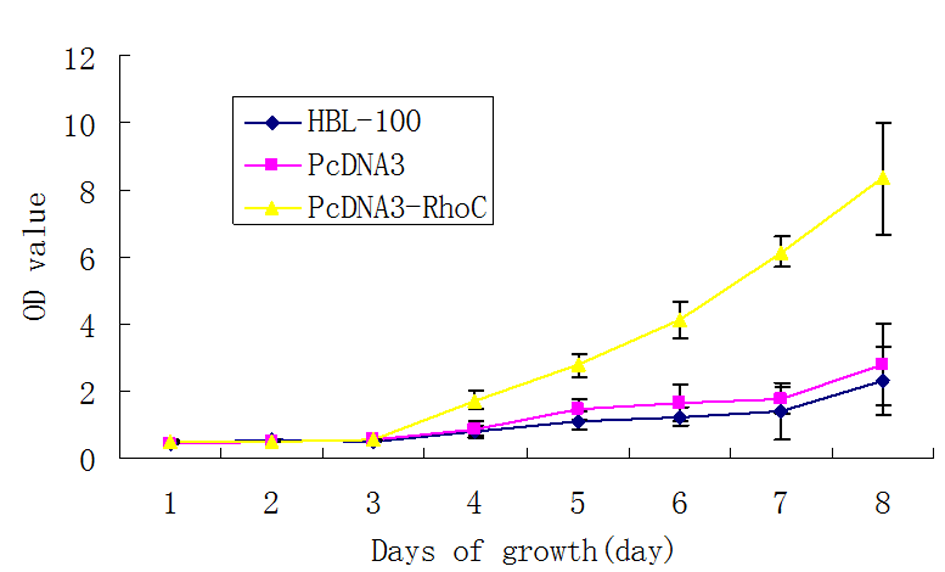

Supplement: Figure S1 — Growth curve of human breast epithelial HBL-100 cells transfected with empty vector or RhoC. Cells without transfection were applied as control. (TIF) [file pone.0054493.s001.tif]

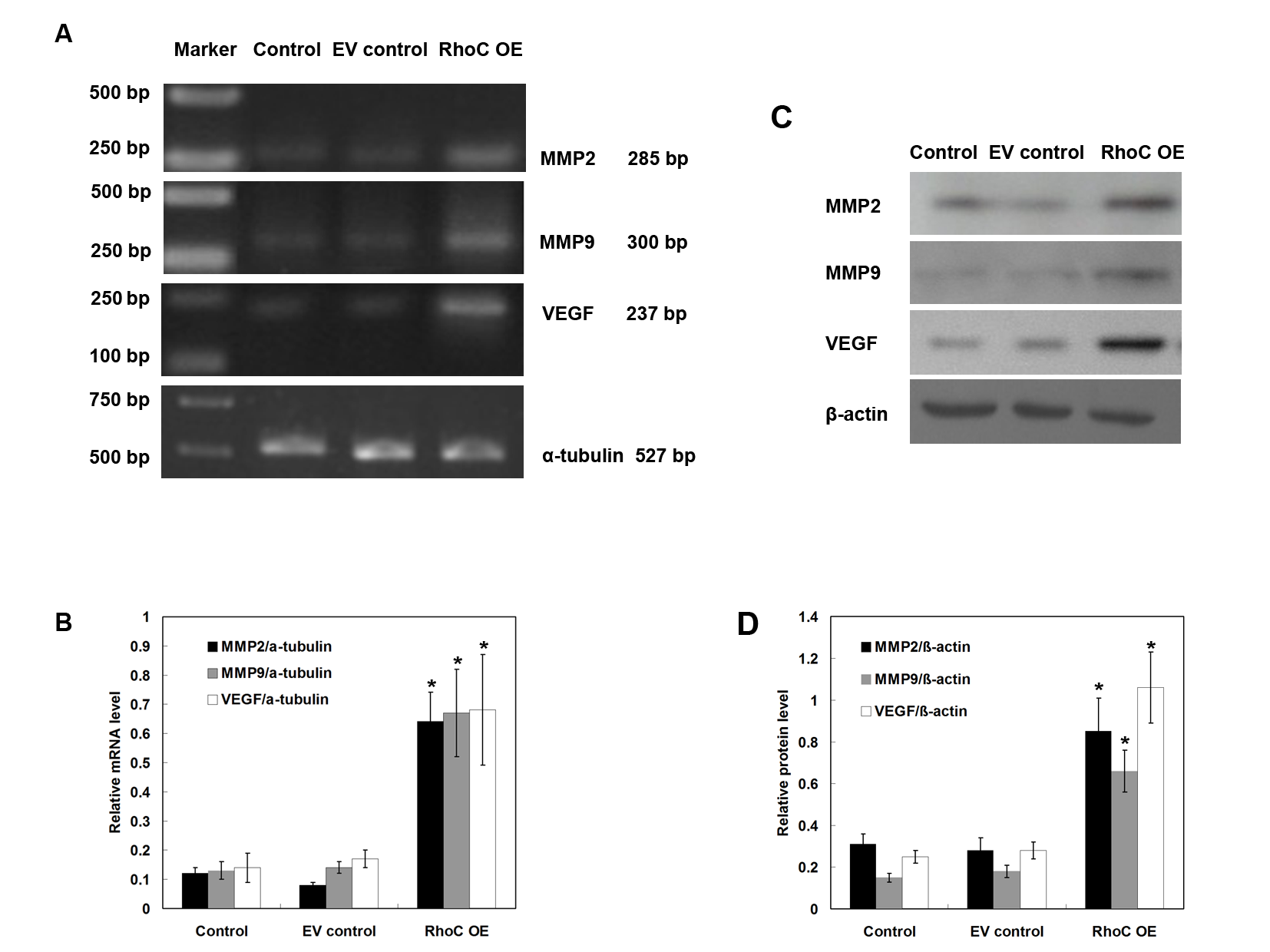

Supplement: Figure S2 — Effects of RhoC overexpression on the regulation of cell migration- and invasion-related gene expressions in human breast epithelial HBL-100 cells. (A) RT-PCR. The expression of MMP2, MMP9 and VEGF mRNA in HBL-100 cells was determined by RT-PCR. α-tubulin was used as an internal control. (B) Quantitative data. The relative mRNA expression of target gene was quantified from three independent experiments. (C) Western blotting. The expression of MMP2, MMP9 and VEGF proteins in HBL-100 cells was determined by Western blotting. β-actin was used as an internal control. (D) Quantitative data. The relative protein expression of target gene was quantified from three independent experiments. *P<0.05 compared with EV control. (TIF) [file pone.0054493.s002.tif]
